# Transparent Wood Fiber-Reinforced Epoxy-Resin Electromagnetic-Shielding Materials with Superior Mechanical Strength and Thermal Insulation Performance

**DOI:** 10.3390/ma18184262

**Published:** 2025-09-11

**Authors:** Jingshu Gao, Zhen Wu, Ling Zhu, Yue Gao, Liping Cai, Zunling Zhu, Yaoli Zhang

**Affiliations:** 1 College of Art and Design, Nanjing Forestry University, Nanjing 210037, China; gjs@njfu.edu.cn (J.G.); 2111310918@njfu.edu.cn (Z.W.);; 2 College of Furnishings and Industrial Design, Nanjing Forestry University, Nanjing 210037, China; 3 School of Economics and Management, Huainan Normal University, Huainan 232001, China; 4 Department of Mechanical Engineering, University of North Texas, Denton, TX 76207, USA; 5 Jiangsu Co-Innovation Center of Efffcient Processing and Utilization of Forest Resources, International Innovation Center for Forest Chemicals and Materials, College of Materials Science and Engineering, Nanjing Forestry University, Nanjing 210037, China

**Keywords:** wood fiber, transparent composite, epoxy resin, electromagnetic shielding

## Abstract

The development of electromagnetic-shielding materials that not only meet the requirements of electromagnetic shielding but also possess transparency and additional functionalities is a new trend in the field. In this study, delignified wood fibers were used as the base material, which were impregnated in epoxy resin and then reinforced with three types of electromagnetic-shielding fillers: chopped carbon fibers, silicon carbide particles, and nano-silica. The experimental results showed that the resulting wood fiber-reinforced epoxy-resin electromagnetic-shielding transparent material not only exhibited excellent mechanical strength and thermal insulation properties but also achieved high haze and effective electromagnetic-shielding efficiency (greater than 90%) while maintaining a transmittance of approximately 50%. Based on the orthogonal experimental results, the optimal performance of the wood fiber-reinforced epoxy-resin electromagnetic-shielding transparent material was obtained when chopped carbon fibers were used as the electromagnetic-shielding filler component, with an electromagnetic-shielding filler mass fraction of 0.3 wt% and a wood fiber mass fraction of 5.0 wt%.

## 1. Introduction

The rapid advancement of electronic technology has significantly enhanced the convenience of modern life. However, when the electromagnetic-radiation intensity of electronic devices exceeds a critical threshold, it gives rise to electromagnetic pollution. This form of pollution not only compromises the precision and operational reliability of electronic systems but also poses serious risks to human health [1]. Buildings, as essential spaces for human habitation, have become primary environments for electromagnetic pollution. The combined effects of outdoor sources such as 4G/5G base stations, power substations, high-voltage transmission lines, and TV broadcasting towers, together with indoor sources including household appliances, kitchen equipment, and office electronics, have intensified the severity of indoor electromagnetic-radiation exposure [2,3].

To mitigate electromagnetic pollution, it is imperative to enhance regulatory standards and monitoring frameworks for electromagnetic radiation in built environments, while simultaneously advancing the development of protective technologies [2]. Among these, electromagnetic shielding and absorption are core strategies for mitigating radiation hazards. Electromagnetic shielding involves the use of designated materials or structural designs to inhibit the penetration of external electromagnetic fields into protected zones and is widely recognized as the most effective method for radiation suppression. A wide range of shielding materials tailored for architectural applications—such as electromagnetic-shielding concrete, absorbing coatings, shielding glass, and conductive fabrics—have been developed globally [4,5,6,7].

From a materials science perspective, high-performance electromagnetic-shielding materials must exhibit a balanced integration of attributes: lightweight, high shielding effectiveness, durability, ease of fabrication, cost-efficiency, and robust mechanical integrity. In building applications, additional requirements emerge, including elevated mechanical strength and thermal-insulation performance. For components such as windows, skylights, and decorative partitions, maintaining a requisite level of optical transparency is also essential. In this regard, notable progress has been made by researchers and engineers [6,7,8,9,10]. Nevertheless, the fabrication of composite materials that simultaneously achieve optical transparency and effective electromagnetic shielding remains a considerable challenge—particularly when deployed in structural contexts where mechanical robustness and thermal performance are non-negotiable.

Transparent wood has emerged as a novel and promising multifunctional material platform, attracting considerable interest due to its sustainability, low cost, favorable optical properties, excellent mechanical strength, and low thermal conductivity [8,9,10,11,12,13]. Zhang et al. [8] comprehensively reviewed the structural features, chemical composition, and performance-determining factors of transparent wood. Recent research has demonstrated the feasibility of endowing transparent wood with electromagnetic-shielding capabilities [14,15,16,17,18,19]. Methods explored include the application of indium tin oxide (ITO) coatings and the incorporation of magnetic nanoparticles such as Fe_3_O_4_ to enhance shielding efficiency [17,18]. Furthermore, through structural and compositional optimization, the electromagnetic-shielding performance can be further improved, positioning transparent wood as a sustainable alternative for electromagnetic-shielding applications [18]. However, current fabrication techniques often rely on delignified wood templates, which restrict sample sizes to millimeter or centimeter scales—thereby limiting their broader applicability.

The present study builds on the author’s previous work in transparent wood-fiber-material development [19], which successfully replaced bulk wood boards with lignin-depleted wood fibers to fabricate large-area transparent composites with improved functional performance [19,20]. In this work, three types of electromagnetic-shielding fillers—short carbon fibers (C), silicon carbide (SiC) particles, and titanium dioxide (TiO_2_) nanoparticles—were incorporated into an epoxy-resin matrix reinforced with wood fibers to fabricate fiber-reinforced epoxy-based electromagnetic-shielding transparent wood (FEESTW). Using orthogonal experimental design, the study systematically investigated the effects of filler type, filler content, and wood fiber content on electromagnetic-shielding effectiveness, as well as the optical, thermal, mechanical, and acoustic properties of the composite material. The goal was to develop a series of multifunctional, high-performance, transparent wood-based electromagnetic-shielding materials suitable for a range of applications including architecture, packaging, and energy-saving systems. These materials can regulate electromagnetic radiation within acceptable limits while fulfilling structural and functional requirements, thus enabling effective control of electromagnetic environments and safeguarding human health and well-being.

## 2. Experiment

### 2.1. Experimental Materials

To optimize the overall performance characteristics, wood fiber-reinforced epoxy-resin transparent composites were fabricated utilizing 40-mesh wood fibers as the reinforcing phase. The polymer matrix consisted of carboxyl-terminated bisphenol A epoxy resin (Nanjing Biochemical Reagent Co., Ltd., Nanjing, China). The electromagnetic-shielding fillers comprised short carbon fibers with a diameter of 7 μm and length of 0.5 mm (Carbon Tech Co., Ltd., Shenzhen, China), silicon carbide (SiC) particles (Shanghai Pantian Powder Material Co., Ltd., Shanghai, China), and titanium dioxide (TiO_2_) nanoparticles with particle sizes of <5 nm (Aladdin Biochemical Technology Co., Ltd., Shanghai, China). Additional reagents included anhydrous ethanol and deionized water, both of analytical grade.

### 2.2. Preparation Method

**Figure 1:**
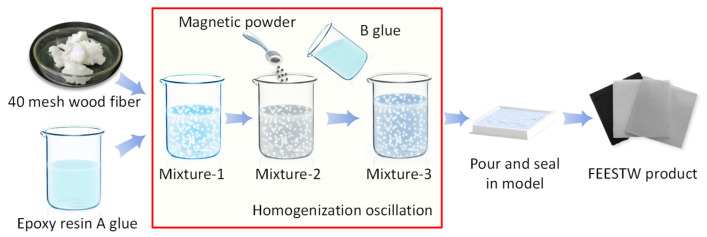
Preparation process of fiber-reinforced epoxy-resin electromagnetic-shielding transparent wood.

The fabrication process of wood fiber-reinforced epoxy-resin electromagnetic-shielding transparent composites is illustrated in Figure 1 and detailed as follows:

Figure 1 shows the preparation process of fiber-reinforced epoxy-resin electromagnetic-shielding transparent wood composites:

(1) A predetermined quantity of wood powder fibers was accurately weighed using a precision electronic balance and immersed in anhydrous ethanol for subsequent processing.
(2) The ethanol was carefully expelled, leaving the wood fibers in a slightly damp state. The fibers were then thoroughly mixed with the corresponding proportion of epoxy resin component A through mechanical stirring with a glass rod, followed by ultrasonic treatment to ensure homogeneous dispersion.
(3) The resulting mixture was placed in a vacuum drying oven and evacuated for 10 min to achieve complete ethanol evaporation.
(4) A predetermined amount of electromagnetic-shielding filler powder and the corresponding proportion of curing agent B were incorporated into the mixture. The components were thoroughly blended using mechanical stirring and ultrasonic treatment to ensure uniform distribution throughout the matrix.
(5) The homogenized mixture was cast into a designated mold and placed in a vacuum drying oven. Curing was performed at 45 °C for 6 h to obtain the final wood fiber-reinforced epoxy-resin electromagnetic-shielding transparent composite.

Based on this preparation methodology, the present study systematically investigated the influence of electromagnetic-shielding filler composition, filler mass fraction, and wood fiber content on the properties of the resulting composites. An orthogonal experimental design was implemented to evaluate the effects of electromagnetic-shielding filler components, primarily carbon fibers and SiC particles, while incorporating the functional properties of TiO_2_ nanoparticles. The parameter levels for electromagnetic-shielding filler composition, filler mass fraction, and wood fiber content are summarized in Table 1, with the corresponding experimental matrix presented in Table 2.

**Figure 2:**
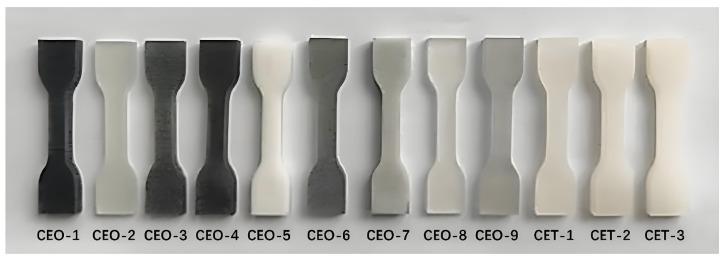
FEESTW samples prepared with different parameters.

Figure 2 presents representative samples of 12 wood fiber-reinforced epoxy-resin electromagnetic-shielding transparent composites, corresponding to the experimental conditions outlined in Table 2. The results demonstrate that preparation parameters exert significant influence on material homogeneity and pore structure, consequently affecting sample performance characteristics. Subsequent quantitative analysis systematically evaluates these parametric effects.

### 2.3. Performance Characterization Methods

**Figure 3:**
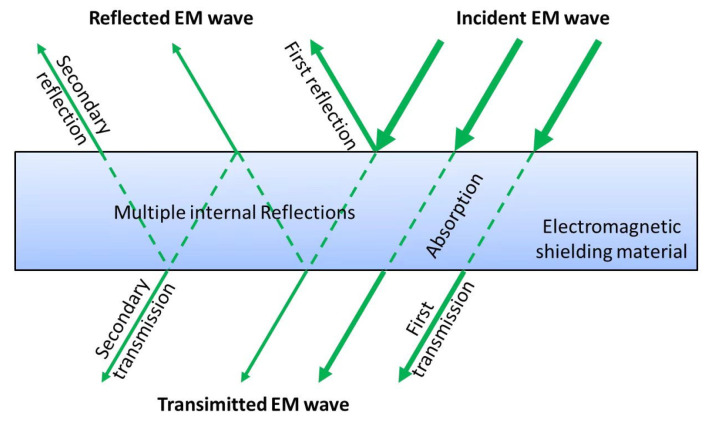
Principle of material electromagnetic shielding.

The comprehensive characterization of wood fiber-reinforced epoxy-resin electromagnetic-shielding transparent composites encompassed material morphology and microstructure, electromagnetic wave-absorption properties, electromagnetic-shielding effectiveness, optical properties, thermal behavior, and mechanical performance. The fundamental principle of electromagnetic-wave absorption and shielding is illustrated in Figure 3. When incident electromagnetic waves interact with the shielding material, the electromagnetic energy undergoes partitioning into three distinct components [7]: (1) surface reflection at the material interface, (2) multiple internal reflections and absorption within the material matrix, and (3) residual electromagnetic-wave transmission through the material. Throughout these interaction processes, electromagnetic-wave energy attenuation occurs through three primary mechanisms: reflection loss (surface reflection), absorption loss (dielectric and magnetic losses), and multiple reflection loss (internal scattering and re-reflection phenomena).

The electromagnetic-shielding performance was quantitatively evaluated using the shielding effectiveness (SE). The total shielding effectiveness (SEtotal) can be expressed as follows [21]:

(1) $$\mathrm{SEtotal}=10\log_{10}\left(P_{1}/P_{2}\right)$$

In this context, *P*_1_ and *P*_2_ represent the power density functions of the incident wave and transmitted wave, respectively.

The absorption performance can be characterized by the reflection loss (*RL*), which is defined as the difference between the incident power and the reflected power [22]:

(2) $$RL=10\log_{10}\left(P_{R}/P_{1}\right)$$

*P_R_* represents the power density function of the reflected wave.

During the testing process, following the GJB 2038A-2011 “Method for Reflectivity Testing of Radar Absorbing Materials”, the electromagnetic properties of the material were measured using an E5061A Vector Network Analyzer (Keysight Technologies, Santa Rosa, CA, USA). The NRW method was employed to determine the complex permittivity *ε* and permeability *µ* of the tested samples. Subsequently, the reflection loss (*RL*), wave impedance (Zin), and electromagnetic-wave attenuation constant (*α*) were calculated [22]. These parameters were used to determine the electromagnetic-shielding effectiveness of the material:

(3) SE=A+R+M

*A* represents absorption loss, *R* represents reflection loss, and *M* represents multiple reflection loss. Absorption loss was generated by induced eddy currents:

(4) $$A=20\log_{10}\left[\exp(-\alpha t)\right]=-8.68\alpha t$$

*t* represents the thickness of the material, and *α* represents the electrical conductivity.

Multiple reflection loss was generated as electromagnetic waves underwent successive reflections and transmissions through the shielding material, and its magnitude is as follows [23]:

(5) $$M=20\log_{10}\left|1-{\Gamma_{1}}^{2}e^{-2\alpha t}\right|$$

$\Gamma_{1}$ represents the reflection coefficient, which satisfies the following relationship [23]:

(6) $${\Gamma_{1}}^{2}=1-1/10^{-RL/20}$$

The frequency range of electromagnetic waves is vast, spanning over twenty orders of magnitude, from extremely low frequencies to high-energy radiation. This article focused on the X-band electromagnetic waves, which are widely used in space research, communication broadcasting, meteorological satellites, and other applications. The frequency range of the X-band was 8.0 to 12.4 GHz. The corresponding test sample size was 10.2 × 22.9 × 1.5 mm.

The characterization methods and literature references for optical properties, thermal properties, and mechanical properties are consistent as stated in reference [19]. The microstructure of the sample was observed using a JSM-6700F field emission scanning electron microscope. The optical performance of the sample mainly focuses on its transparency and haze, which are measured using a Hitachi U-4100 spectrophotometer (Hitachi, U-4100, Tokyo, Japan) according to ASTM D1003 “Standard Method for Haze and Transmittance of Transparent Plastics”. The thermal conductivity is measured using the heat flow meter method according to the requirements of GB/T 10294 and is measured using an EKO HC-074-200 thermal conductivity meter from the United States. The mechanical properties are tested using a multifunctional mechanical testing machine (AGS-X) in accordance with “GB/T 1041-2008 Determination of Plastic Compression Properties”.

## 3. Results

This study primarily investigated the microstructural characteristics, fundamental properties (optical, thermal, and mechanical properties), and electromagnetic-shielding effectiveness of wood fiber-reinforced epoxy-resin transparent composites. Through electron microscopy analysis, the interfacial relationships between wood fibers and electromagnetic-shielding fillers, as well as their influence on material properties, were elucidated. Range analysis was employed to determine the primary and secondary factor relationships governing material properties and their optimal parameter combinations.

**Figure 4:**
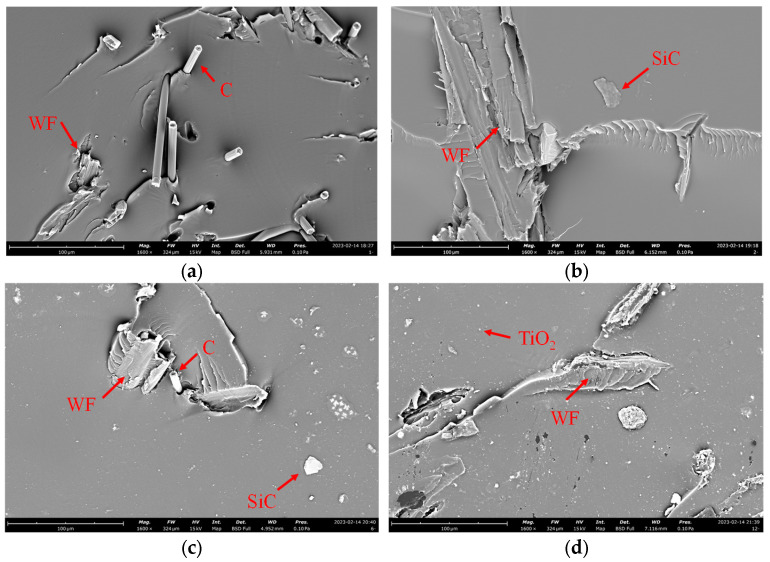
SEM images of FEESTW samples. (**a**) Typical C-containing powder materials (CEO-1). (**b**) Typical SiC-containing powder materials (CEO-2). (**c**) Typical CEO-3-containing powder materials (CEO-3). (**d**) Typical TiO_2_-containing powder materials (CET-1). WF: wood Fiber.

**Figure 5:**
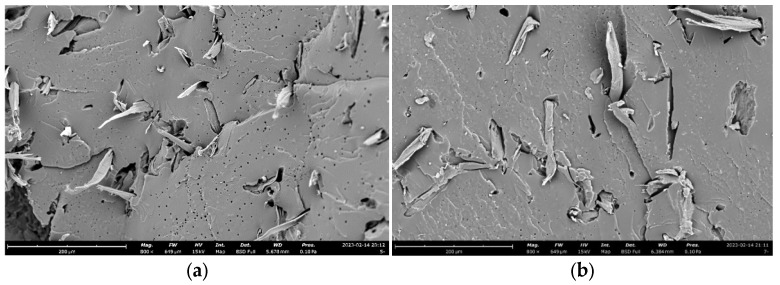
SEM images of low transparency FEESTW samples. (**a**) CEO-5, (**b**) CEO-7.

Figure 4 presents typical SEM micrographs of wood fiber-reinforced epoxy-resin electromagnetic-shielding transparent composites containing C, SiC, C-SiC, and TiO_2_ electromagnetic-shielding fillers. The carbon powder exhibited a short-fiber morphology, and owing to the substantially higher mechanical strength of carbon fibers relative to the polymer matrix, they displayed characteristic fiber pull-out behavior at the interface, indicating favorable interfacial adhesion between carbon fibers and the epoxy matrix. SiC particles appeared as discrete entities uniformly dispersed within the polymer matrix, while TiO_2_ nanoparticles demonstrated fine particle size distribution and homogeneous dispersion throughout the epoxy-resin matrix. Figure 5 shows the SEM micrographs of CEO-5 and CEO-7. The samples of both formulations showed significant mixing effects, and there were obvious interface defects between the matrix and the doped phase, which also had a significant impact on their performance. We will further analyze this feature in the following text.

### 3.1. Structure and Performance Characterization

**Figure 6:**
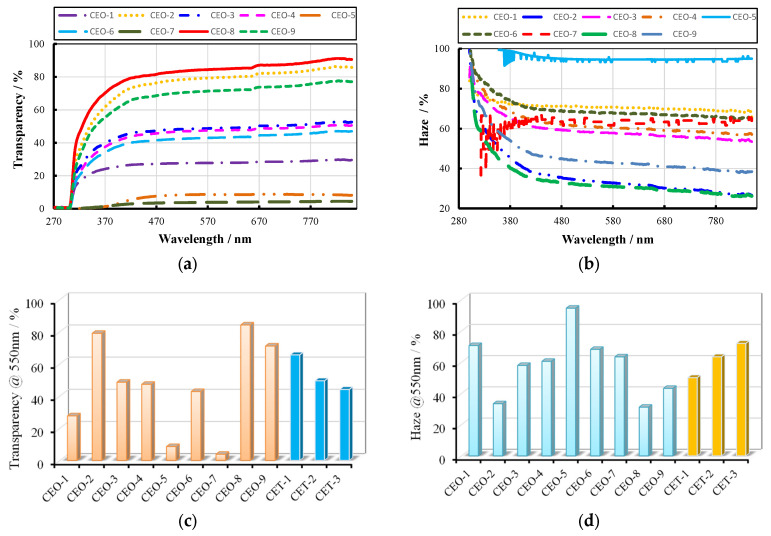
Typical optic performance of FEESTW samples prepared with different parameters. (**a**) Luminousness, (**b**) haze, (**c**) transparency at 550 nm, (**d**) haze at 550 nm.

The characteristic optical properties of wood fiber-reinforced epoxy-resin electromagnetic-shielding transparent composites are illustrated in Figure 6. The optical performance of samples prepared under different parametric conditions exhibited consistent spectral trends with varying wavelengths, while demonstrating significant quantitative differences. Transmittance and haze generally displayed an inverse correlation, with higher transmittance corresponding to reduced haze values, although specific samples (CEO-5 and CEO-7) deviated from this conventional relationship. Comparative analysis of optical properties revealed that CEO-5 and CEO-7 exhibited substantially lower transmittance relative to other samples, accompanied by anomalous haze characteristics. This phenomenon was primarily attributed to elevated wood fiber content in these composites, which promoted bubble formation during the curing and consolidation process (as depicted in Figure 5. The presence of these voids increased the optical scattering cross-section, resulting in diminished transmittance and enhanced haze. This porous microstructural feature concurrently influenced other material properties, which are systematically analyzed in subsequent sections.

Figure 6a,b illustrate the transmittance and haze of 12 different material types at a wavelength of 550 nm, enabling a direct comparison of their optical-performance parameters at this specific wavelength. The range analysis results of the C-SiC-wood fiber orthogonal experiment are presented in Table 3. According to the analysis, the wood fiber mass fraction exerted the most significant influence on transmittance, followed by the type of electromagnetic-shielding filler and its mass fraction. The optimal parameter combination for maximizing transmittance was identified as a wood fiber mass fraction of 5 wt.%, a SiC electromagnetic-shielding filler, and a filler mass fraction of 0.1 wt.%. In terms of haze, the most influential factor was the electromagnetic-shielding filler mass fraction, followed by the wood fiber mass fraction and the filler type. The combination that yielded the highest haze consisted of a 0.3 wt.% electromagnetic-shielding filler mass fraction, a 10 wt.% wood fiber mass fraction, and a C-type filler. Taking both transmittance and haze into consideration, the optimal overall performance was achieved by sample CEO-3, which exhibited a transmittance of 48.6% and a haze value of 57.9%.

Additionally, Figure 6c,d depicts the transmittance and haze of samples with varying TiO_2_ content. A clear trend is observed: as the TiO_2_ mass fraction increases, transmittance decreases while haze increases. When the TiO_2_ mass fraction reached 0.3 wt.%, the material exhibited relatively balanced optical performance, slightly surpassing that of the C-SiC-wood fiber system.

**Figure 7:**
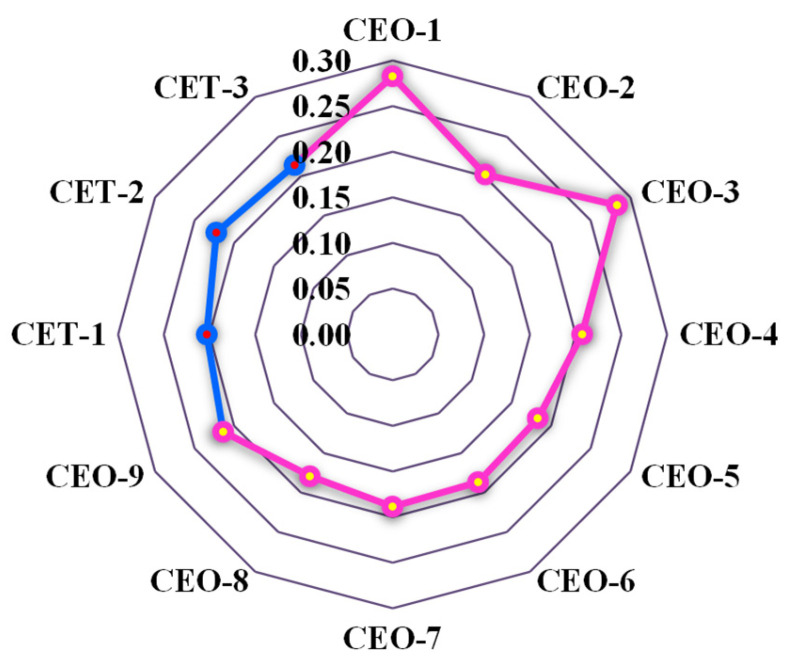
Typical thermal performance of FEESTW samples prepared with different parameters.

**Figure 8:**
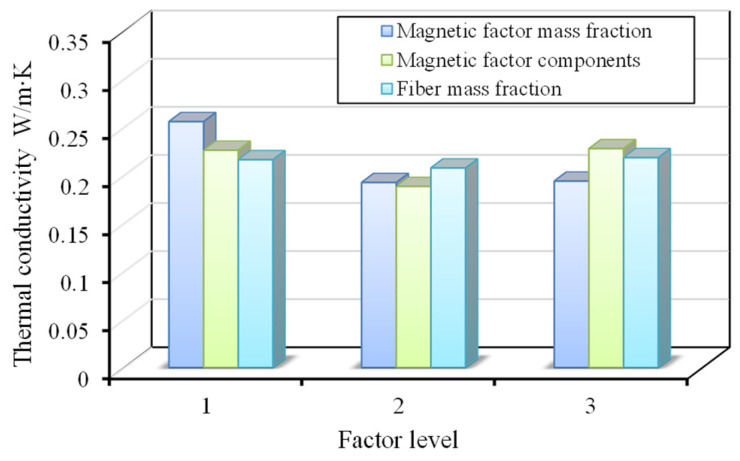
Relationship between factor level and thermal conductivity.

The typical thermal properties of wood fiber-reinforced epoxy-resin electromagnetic-shielding transparent materials are shown in Figure 7, with the thermal conductivity of the nine C-SiC orthogonal experimental materials ranging from 0.179 to 0.283 W/m∙K. The relationship between factor levels and thermal conductivity is shown in Figure 8. Through range analysis, it can be concluded that the order of importance of the factors affecting thermal conductivity is as follows: electromagnetic-shielding filler mass fraction, type of electromagnetic-shielding filler, and wood fiber mass fraction. Looking at the types of electromagnetic-shielding fillers, chopped carbon fibers have a greater influence on thermal conductivity, which is related to the fact that carbon fibers are good thermal conductors. The parameter combination for the best thermal insulation performance is as follows: electromagnetic-shielding filler mass fraction of 0.3 wt%, type of electromagnetic-shielding filler as SiC, and wood fiber mass fraction of 5 wt%. When the electromagnetic-shielding filler is TiO_2_, the thermal conductivity ranges from 0.203 to 0.223 W/m∙K, with the highest thermal conductivity at a TiO_2_ mass fraction of 0.3 wt%. Compared with non-electromagnetic-shielding wood fiber epoxy-resin transparent materials, the addition of the three types of electromagnetic-shielding fillers results in minimal differences in thermal conductivity, all maintaining a low level of around 0.2 W/m∙K, indicating good thermal insulation characteristics.

**Figure 9:**
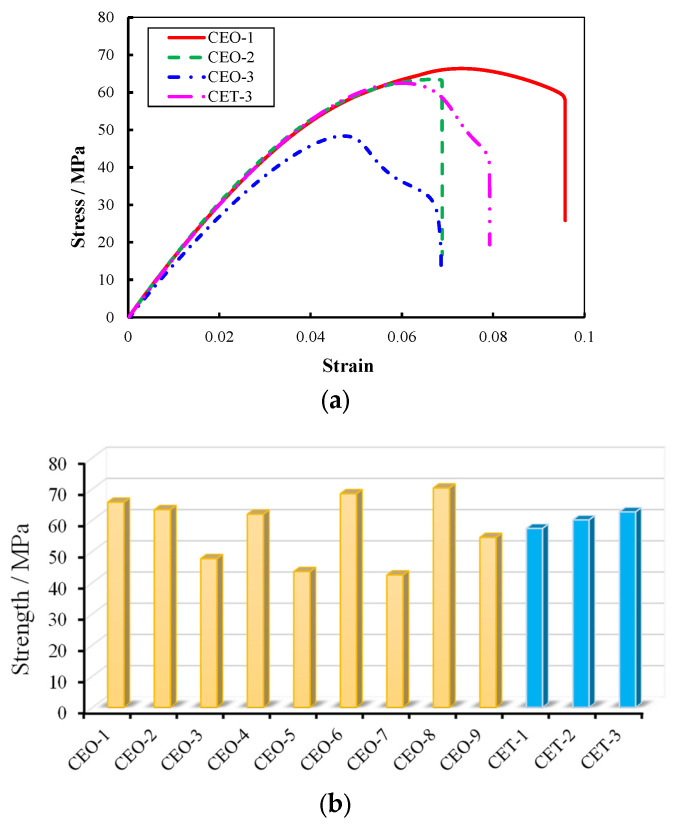
Typical mechanical performance of FEESTW samples prepared with different parameters. (**a**) Typical stress–strain curve, (**b**) material strength.

The typical mechanical properties are presented in Figure 9a,b, where the tensile strength of the materials ranges from 40 to 70 MPa. The results of the C-SiC orthogonal experiment revealed significant variation across the samples. A notable enhancement in strength was observed in materials containing electromagnetic-shielding fillers compared with those without, effectively addressing the issue of “virtual contact” at the interface between the wood fiber reinforcement and the epoxy-resin matrix caused by their differing polarities. This interfacial improvement substantially increased the overall material strength.

As shown in Table 4, range analysis of tensile strength indicated that the most influential factor was the wood fiber mass fraction, followed by the type of electromagnetic-shielding filler and its mass fraction. The optimal parameter combination for maximizing strength was identified as a wood fiber mass fraction of 2.5 wt.%, a short-cut carbon fiber filler, and a filler mass fraction of 0.3 wt.%.

Figure 9 also illustrates the mechanical properties of TiO_2_-reinforced materials, which exhibited tensile strengths in the range of 50–60 MPa, increasing with higher TiO_2_ content. This improvement is attributed to the nanoscale TiO_2_ particles enhancing interfacial adhesion between the fibers and the epoxy matrix.

### 3.2. Analysis of Absorption Performance

**Figure 10:**
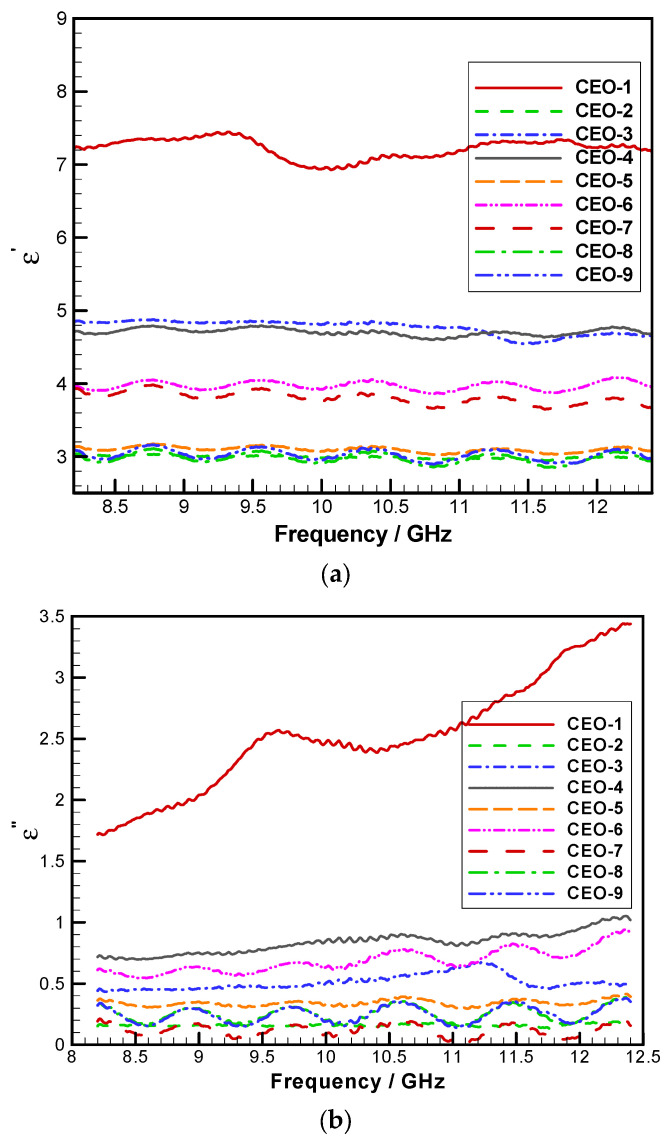
Dielectric constant of FEESTW samples. (**a**) Real part of the dielectric constant, (**b**) imaginary part of the dielectric constant.

The dielectric properties of C-SiC electromagnetic-shielding filler-modified wood fiber-reinforced epoxy-resin transparent materials are illustrated in Figure 10. The real part of the dielectric constant reflects the material’s capacitance, indicating its capacity to store electric energy under an external electric field. In contrast, the imaginary part represents dielectric loss—commonly referred to as the loss factor—which quantifies the dissipation of electric energy into the external field. A higher value of the imaginary component signifies greater electrical conductivity and, consequently, lower insulation performance.

**Figure 11:**
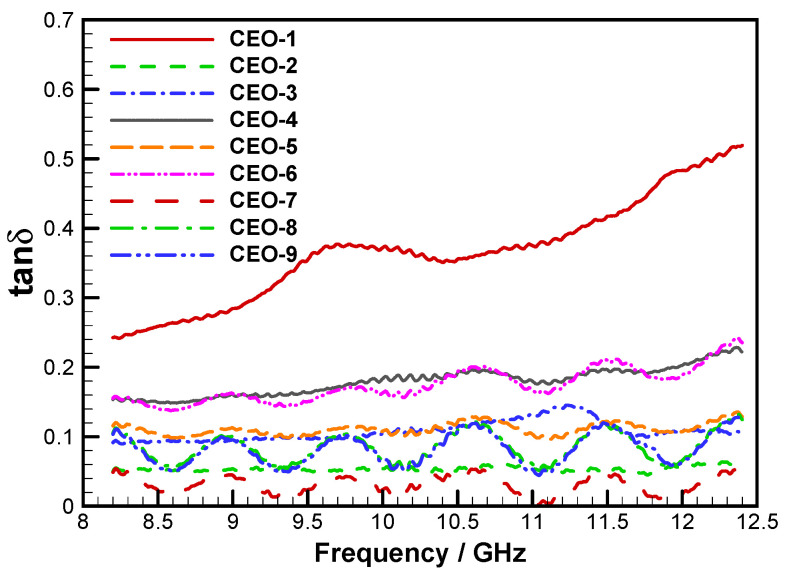
Dielectric loss angle tangents of FEESTW samples.

The ratio of the imaginary to the real part of the dielectric constant, known as the loss tangent, reflects the material’s ability to couple with incident microwaves. A higher loss tangent denotes stronger microwave-absorption capability. Figure 11 presents the loss tangent values for the nine groups of samples evaluated in the orthogonal experiment.

According to the test results, the real part of the dielectric constant exhibited minor fluctuations across the frequency range, indicating good overall stability. Meanwhile, both the imaginary part and the loss tangent showed a slight increase with rising electromagnetic-wave frequency. When comparing the dielectric performance of materials fabricated under different parameter combinations, sample CEO-1 demonstrated the most favorable dielectric characteristics, with the highest values for the real part, imaginary part, and loss tangent. This was followed by CEO-3 and CEO-4, which corresponded to the second-highest mass fractions of short-cut carbon fibers. These results suggest that short-cut carbon fibers were the dominant factor affecting the dielectric behavior in the C-SiC orthogonal experiment.

In the subsequent analysis, a more quantitative assessment will be conducted, focusing on reflection loss, electromagnetic-wave attenuation constant, and overall electromagnetic-shielding effectiveness.

**Figure 12:**
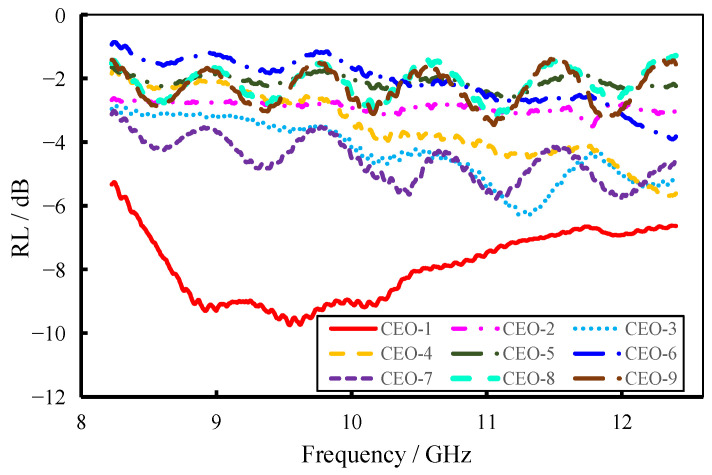
Reflection-loss curves of FEESTW samples.

**Figure 13:**
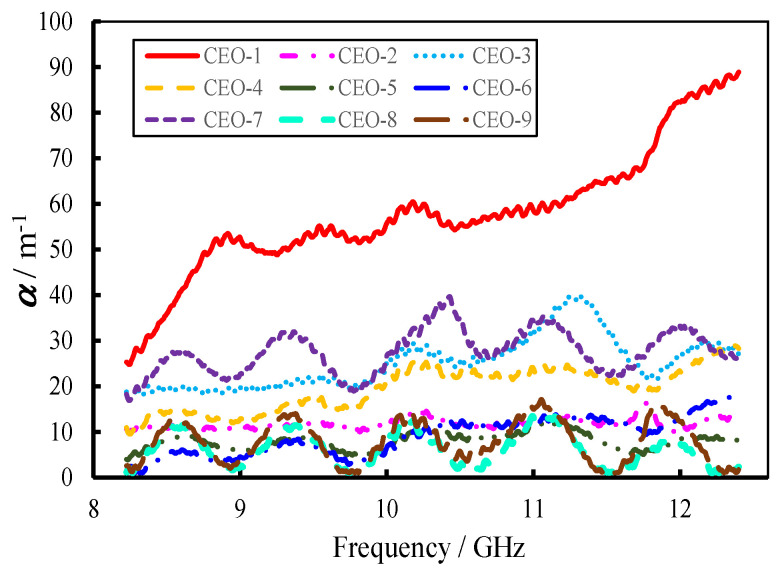
Attenuation constant of FEESTW samples.

The reflection loss and electromagnetic-wave attenuation constant of the materials were calculated using the computational methods outlined in Section 2.3. The corresponding results are presented in Figure 12 and Figure 13, with a fixed sample thickness of 3 mm used for all calculations. The data indicate that although the reflection loss of the C-SiC orthogonal experiment materials was relatively limited, the electromagnetic-wave attenuation constant was considerably high. Among the nine tested samples, CEO-1 exhibited the most pronounced performance in both reflection loss and attenuation constant. Specifically, its reflection loss reached a minimum value of –9.74 dB at 9.56 GHz. For frequencies above 9 GHz, the attenuation constant surpassed 50 m^−1^ and continued to increase with frequency, albeit with slight fluctuations.

**Figure 14:**
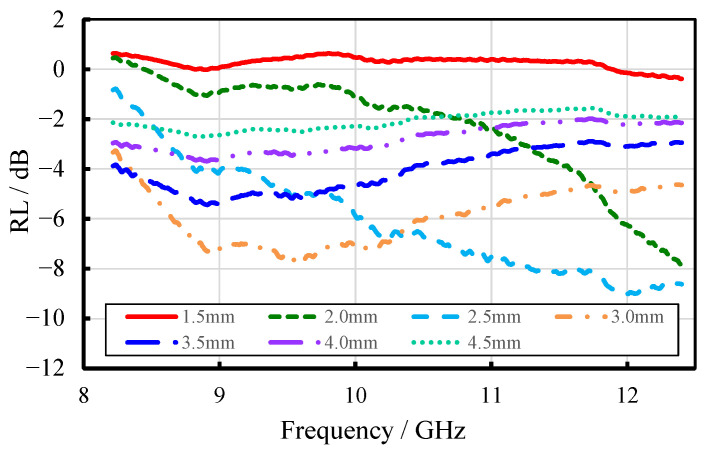
Reflection-loss curves of different FEESTW samples with different thicknesses.

Figure 14 illustrates the reflection loss of wood fiber-reinforced epoxy-resin electromagnetic-shielding transparent materials at varying sample thicknesses. The results reveal that, as thickness increased, the minimum reflection loss initially decreased and then increased, while the corresponding frequency of minimum reflection loss shifted from higher to lower values. Optimal absorption performance was observed in samples with thicknesses ranging from 2.5 to 3.0 mm.

This shift in peak reflection-loss frequency can be interpreted through the quarter-wavelength cancelation theory [24,25], in which the sample thickness *d* and the frequency *f_m_* corresponding to the reflection-loss minimum are related by the following equation:

(7) $$f_{m}=\frac{c}{\left[4d\sqrt{\mu_{r}\varepsilon_{r}}\left(1+\tan^{2}\frac{\delta }{8}\right)\right]}$$

where $\mu_{r}$ and $\varepsilon_{r}$ are the complex permittivity and complex permeability, *c* is the speed of light in vacuum, and tanδ is the loss tangent of the dielectric. It is evident that for the same material, $\mu_{r}$, $\varepsilon_{r}$tanδ remained consistent, and as the thickness of the material increased, the corresponding fm decreased.

**Figure 15:**
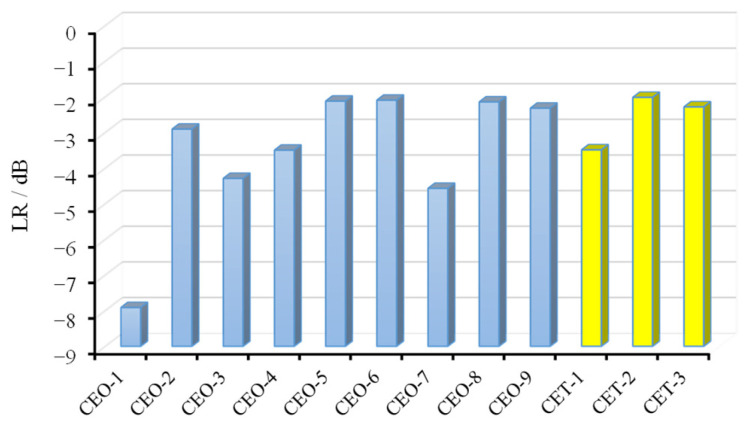
Mean reflection loss of different FEESTW samples.

**Figure 16:**
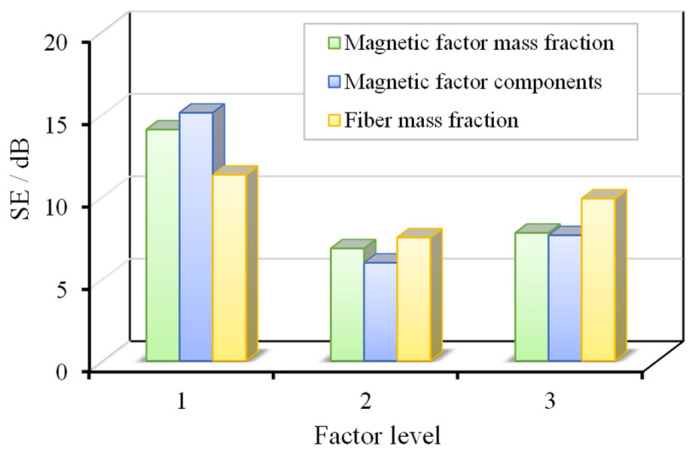
Relationship between factor level and mean reflection loss.

The average reflection loss across the tested frequency range was calculated, as shown in Figure 15. To further investigate the factors influencing the absorption performance of wood fiber-reinforced epoxy-resin electromagnetic-shielding transparent materials, an extreme difference analysis was conducted based on this parameter, with the results presented in Figure 16. The primary factors affecting the absorption performance of the C-SiC-epoxy-resin composite materials, in descending order of influence, were identified as the type of electromagnetic-shielding filler, the filler mass fraction, and the wood fiber mass fraction.

The optimal absorption performance was achieved when the electromagnetic-shielding filler was short-cut carbon fibers, the filler mass fraction was 0.5 wt.%, and the wood fiber mass fraction was 2.5 wt.%. Furthermore, the computational results indicate that short-cut carbon fibers contributed substantially more to absorption performance compared with SiC and TiO_2_. In contrast, the effect of TiO_2_ mass fraction on absorption performance was relatively limited, with the best performance observed at a TiO_2_ content of 0.5 wt.%.

### 3.3. Electromagnetic Shielding Effectiveness Analysis

**Figure 17:**
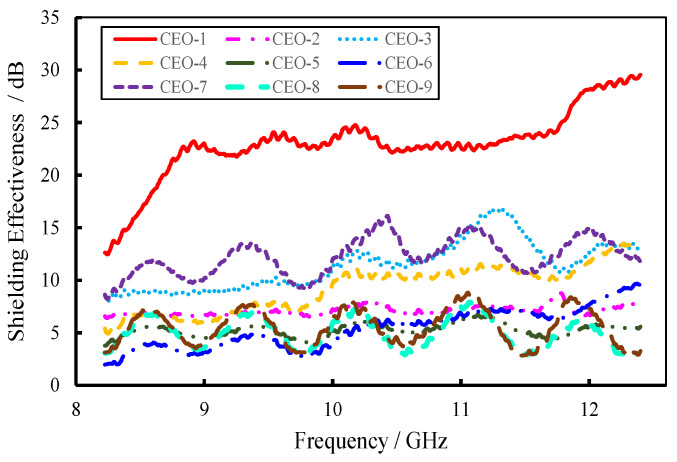
Electromagnetic-shielding effectiveness of FEESTW samples.

**Figure 18:**
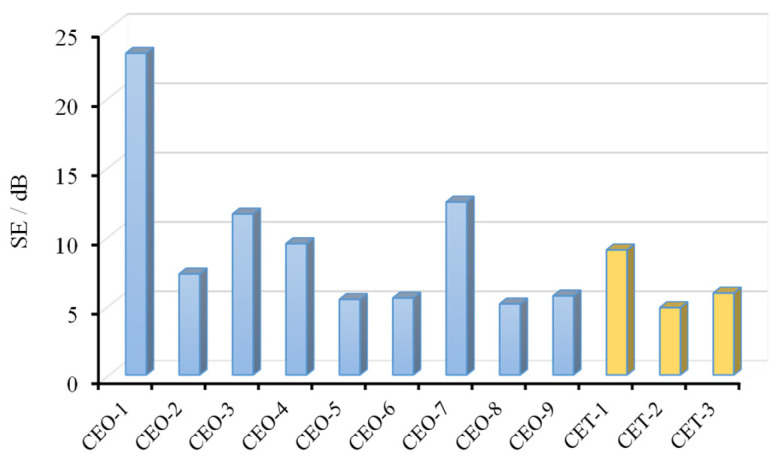
Electromagnetic-shielding effectiveness of 12 FEESTW samples.

The electromagnetic-shielding effectiveness (EMSE) was calculated using Equation (1), and the results are shown in Figure 17. The C-SiC-epoxy-resin orthogonal experiment materials exhibited significant variation in EMSE. A quantitative evaluation was carried out by calculating the average shielding effectiveness across the tested frequency range, as illustrated in Figure 18. The EMSE values for the nine groups of C-SiC-epoxy-resin composites ranged from 5.1 dB to 23.2 dB, corresponding to shielding efficiencies between 69.2% and 99.5%.

These results demonstrate that, even under controlled conditions where the mass fraction of electromagnetic-shielding fillers was limited to preserve transparency and haze, the materials still effectively attenuated electromagnetic radiation. This indicates their potential as architectural materials capable of mitigating radiation exposure and safeguarding human health.

Figure 17 also includes the EMSE performance of TiO_2_-enhanced materials. The optimal performance was observed at a TiO_2_ filler mass fraction of 0.5 wt.%, achieving a shielding effectiveness of 9.16 dB.

The results of the extreme difference analysis for electromagnetic-shielding effectiveness (EMSE) are summarized in Table 5. It is evident that the most critical factor influencing EMSE is the type of electromagnetic-shielding filler. The incorporation of short-cut carbon fibers significantly enhanced the shielding performance. The second most influential factor was the mass fraction of the electromagnetic-shielding filler, with higher filler content corresponding to increased shielding effectiveness.

In contrast, the influence of wood fiber content on EMSE was minimal. Within the parameter range investigated in this study, its effect can be considered negligible. The optimal combination of preparation parameters for achieving the highest EMSE was determined to be a 0.5 wt.% electromagnetic-shielding filler mass fraction, short-cut carbon fibers as the shielding component, and a wood fiber mass fraction of 2.5 wt.%.

## 4. Conclusions and Discussion

In this study, a series of wood fiber-reinforced epoxy-resin electromagnetic-shielding transparent materials were fabricated using short-cut carbon fibers, SiC, and nano-TiO_2_ particles as electromagnetic-shielding fillers. A comprehensive investigation was conducted on macroscopic morphology, microscopic structure, optical properties, thermal properties, mechanical properties, absorption performance, electromagnetic-shielding effectiveness, and the underlying shielding mechanisms of these materials. The effects of electromagnetic-shielding filler type, filler mass fraction, and wood fiber mass fraction on the material properties were systematically analyzed.

Based on the orthogonal experimental results, the optimal parameter combinations for various performance metrics are summarized in Table 6. It was found that the primary and secondary influencing factors for electromagnetic-shielding performance and absorption were consistent with the optimized combination derived from the orthogonal design, namely the CEO-1 configuration.

**Figure 19:**
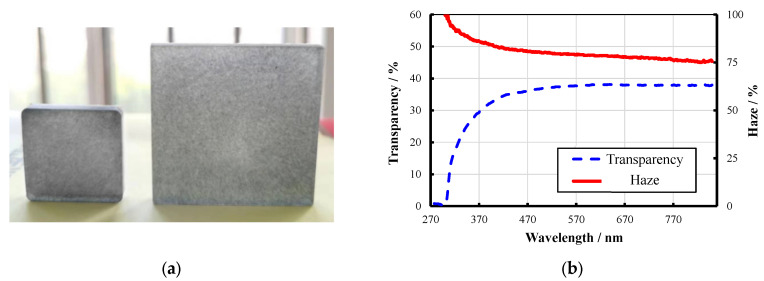
Optimization of haze parameters and its optical properties of samples. (**a**) Haze parameter optimization samples. (**b**) Optical performance of optimization samples.

To further validate the reliability of the orthogonal experiment results, additional samples were prepared with an alternative parameter combination optimized for haze. The selected parameters were 0.3 wt.% electromagnetic-shielding filler mass fraction, short-cut carbon fibers as the filler component, and a 10 wt.% wood fiber mass fraction. Optical performance tests were conducted on these samples, and the fabricated materials along with their optical test results are shown in Figure 19.

The experimental data indicated that the transmittance and haze of the haze-optimized samples at 550 nm were 37.5% and 79.3%, respectively. Notably, the haze value achieved by this optimized scheme exceeded that of the orthogonal experiment, demonstrating that this optimization strategy can effectively enhance haze while maintaining desirable levels of transparency.

In summary, the wood fiber-reinforced epoxy-resin electromagnetic-shielding transparent material prepared in this article has excellent mechanical strength and insulation performance, achieving high haze and effective electromagnetic-shielding efficiency (greater than 90%) while maintaining a light transmittance of about 50%. According to the results of orthogonal experiments, the comprehensive performance of the wood fiber-reinforced epoxy-resin electromagnetic-shielding transparent material is optimal when short-cut carbon fiber is used as the electromagnetic-shielding filler component, the mass fraction of electromagnetic-shielding filler is 0.3 wt%, and the mass fraction of wood fiber is 5.0 wt%. The work of this article extends the functional characteristics of wood fiber transparent wood, clarifies the influence of adding short-cut carbon fibers, SiC, and nano TiO_2_ particles on the electromagnetic-shielding function of wood fiber transparent wood, and obtains performance optimization parameters. Compared with transparent materials with electromagnetic-shielding properties reported in the literature, the material obtained in this paper has significant comprehensive advantages. Compared with the material obtained by Gan [17] adding Fe_3_O_4_ and Cheng et al. [26] composite silver nanowires (AgNW), our material can increase transparency from 28.8% to about 50% in the visible light range, and the longitudinal tensile strength can be increased from 47.8 MPa to 70 MPa. The EMI shielding effect in the X-band can reach 25 dB. At the same time, the material obtained in this paper has low thermal conductivity, a low preparation cost, and easy processing, which can meet the needs of human electromagnetic protection and has broad development prospects.

## Figures and Tables

**Table 1:** Preparation process of fiber-reinforced epoxy-resin electromagnetic-shielding transparent wood.

	Influence Factor	Magnetic Factor Components	Magnetic Factor Mass Fraction [%]	Wood Fiber Mass Fraction [%]
Level				
1		C	0.5	2.5
2		SiC	0.3	5
3		C:SiC = 1:1	0.1	10

**Table 2:** Experiment table of fiber-reinforced epoxy-resin electromagnetic-shielding transparent wood.

	Influence Factor		Magnetic Factor Mass Fraction [%]	Magnetic Factor Components	Wood Fiber Mass Fraction [%]
Experiment Number					
Orthogonal Experiment		CEO-1	1	1	1
		CEO-2	1	2	2
		CEO-3	1	3	3
		CEO-4	2	1	2
		CEO-5	2	2	3
		CEO-6	2	3	1
		CEO-7	3	1	3
		CEO-8	3	2	1
		CEO-9	3	3	2
TiO_2_ supplementary experiment		CET-1	0.1%	TiO_2_	5%
		CET-2	0.3%	TiO_2_	5%
		CET-3	0.5%	TiO_2_	5%

**Table 3:** Range analysis of orthogonal experimental results of FEESTW—transmittance and haze.

Treatment Number	Transmittance			Haze		
	Magnetic Factor Mass Fraction	Magnetic Factor Components	Wood Fiber Mass Fraction	Magnetic Factor Mass Fraction	Magnetic Factor Components	Wood Fiber Mass Fraction
*K* _1j_	155.30	78.80	154.70	162.03	194.64	170.07
*K* _2j_	98.70	171.80	197.40	223.36	159.18	137.19
*K* _3j_	159.10	162.50	61.00	137.82	169.38	215.94
*κ * _1j_	51.77	26.27	51.57	54.01	64.88	56.69
*κ * _2j_	32.90	57.27	65.80	74.45	53.06	45.73
*κ * _3j_	53.03	54.17	20.33	45.94	56.46	71.98
Range	20.13	31.00	45.47	28.51	11.82	26.25
Factor importance	3	2	1	1	3	2
Optimal solution	3	2	2	2	1	3

**Table 4:** Range analysis of orthogonal experimental results of FEESTW—strength.

Treatment Number	Magnetic Factor Components	Magnetic Factor Mass Fraction [%]	Wood Fiber Mass Fraction [%]
*K* _1j_	176.63	169.94	204.37
*K* _2j_	173.74	177.051	179.58
*K* _3j_	167.06	170.44	133.49
*κ * _1j_	58.88	56.65	68.12
*κ * _2j_	57.91	59.02	59.86
*κ * _3j_	55.69	56.82	44.50
Range	3.19	2.37	23.63
Factor importance	2	3	1
Optimal solution	1	2	1

**Table 5:** Range analysis of orthogonal experimental results of electromagnetic-shielding effectiveness.

Treatment Number	Magnetic Factor Mass Fraction	Magnetic Factor Components	Wood Fiber Mass Fraction
*K* _1j_	42.09	45.13	33.87
*K* _2j_	20.46	17.85	22.45
*K* _3j_	23.29	22.86	29.52
*κ * _1j_	14.03	15.04	11.29
*κ * _2j_	6.82	5.95	7.48
*κ * _3j_	7.76	7.62	9.84
Range	7.21	9.09	3.80
Factor importance	2	1	3
Optimal solution	1	1	1

**Table 6:** Comprehensive analysis of performance parameter optimization of FEESTW.

	Optimization Parameters *	Magnetic Factor Mass Fraction	Magnetic Factor Components	Wood Fiber Mass Fraction
Performance Parameters				
Transmittance		3	2	2
Haze		2	1	3
Thermal conductivity		3	2	1
Strength		1	2	1
Reflective loss		1	1	1
Electromagnetic-shielding effectiveness		1	1	1

* Color-Coded Parameter Prioritization: 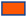 Priority 1, 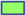 Priority 2, 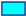 Priority 3, the nu-meric values in the table correspond to the ranking or preferred level for the respective factors.

## Data Availability

The original contributions presented in this study are included in the article. Further inquiries can be directed to the corresponding author.
